# Supplementary material for: Behavioral sensitization and tolerance induced by repeated treatment with ketamine enantiomers in male Wistar rats
Source: PLoS One. 2024 Mar 1;19(3):e0299379. doi: 10.1371/journal.pone.0299379 (PMC10906899; doi:10.1371/journal.pone.0299379)
Supplement: S1 File — (DOCX) [file pone.0299379.s001.docx]

# **Supporting information**

In addition to the presented behavioral effects, we also observed interindividual differences in response to treatment with ketamine enantiomers and racemic ketamine (Fig S1).


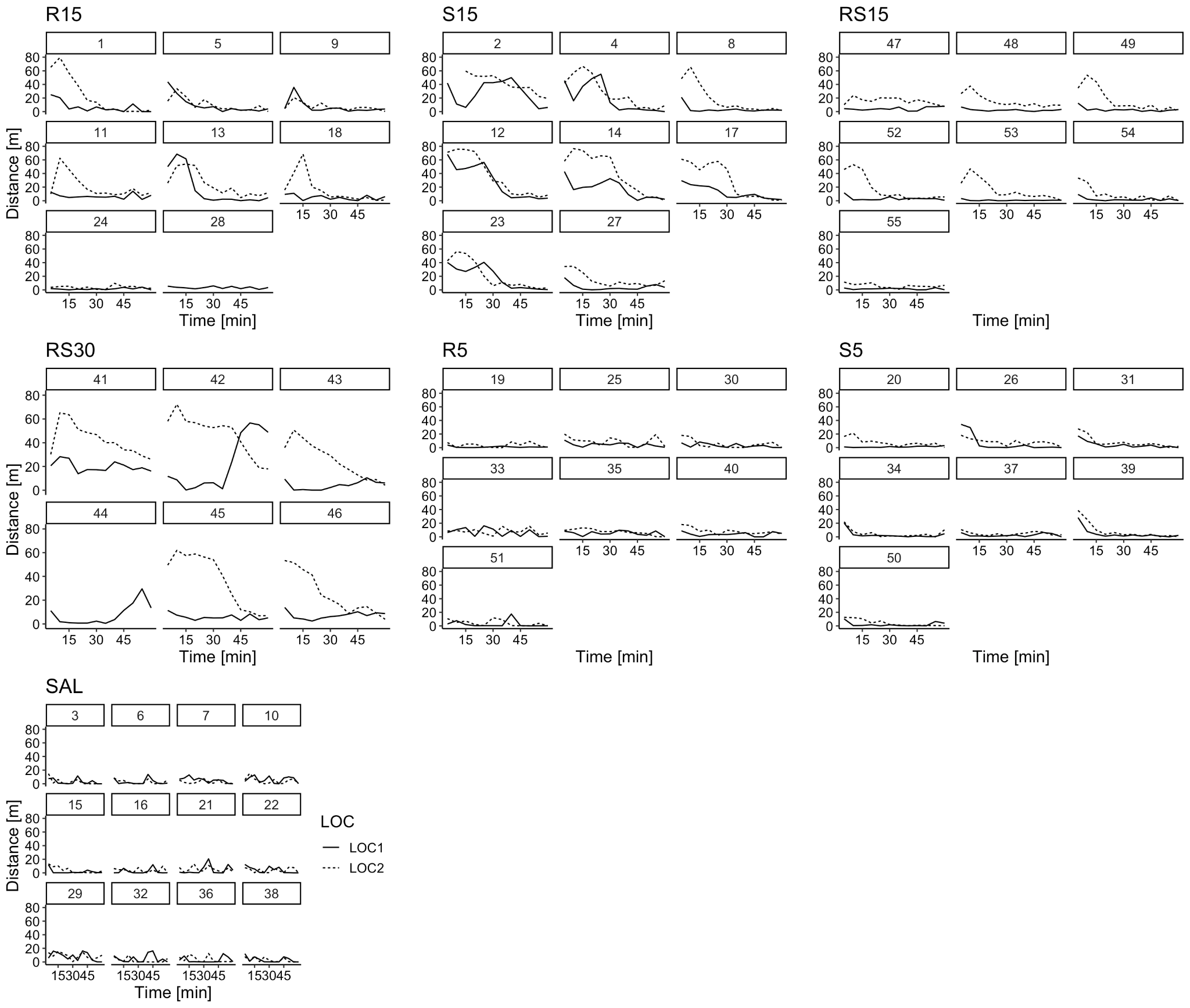


**Fig S1. Interindividual differences in response to treatment with ketamine enantiomers and racemic ketamine.**

Locomotion of individual animals during LOC1 (full line) and LOC2 (dotted line). 5-minute time-bins. R5 - 5 mg/kg of R-ketamine; S5 - 5 mg/kg of S-ketamine; RS15 - 15 mg/kg of racemic ketamine; R15 - 15 mg/kg of R-ketamine; S15 - 15 mg/kg of S-ketamine; RS30 - 30 mg/kg of racemic ketamine; Sal - saline.

During LOC1 (Fig S1, full line), we noticed diverse behavioral effects of the initial S15 treatment among the animals. Out of eight rats, four animals (2, 12, 17, and 23) exhibited stimulated locomotion and ataxia. Animals 4 and 14 displayed the same locomotion patterns but also exhibited dystonia-like behaviors, characterized by lying on the floor, tail twisting, and body twisting. This phase occurred approximately seven minutes after S15 administration and lasted up to ten minutes. Lastly, animals 8 and 27 initially displayed the same behavioral effects as other rats after S-ketamine treatment (stimulated locomotion). However, seven minutes later, they stopped moving and remained stationary. Occasionally, they attempted to move but only made minimal progress with signs of ataxia (falling). Despite their decreased movement, animals 8 and 27 remained awake, occasionally moving their head. Consequently, these two animals exhibited little movement and fewer falls after treatment during LOC1 (the lowest value points of the S15 group during LOC1 in Fig 2 and Fig 4).

In contrast, the interindividual differences in S15 effects were less pronounced during LOC2 after the seventh dose (Fig 4). S15 stimulated locomotion more, did not prevent ambulation, produced fewer ataxic symptoms (occasional falls), and did not lead to dystonia-like behaviors. The slight exception was animal 17; it displayed a comparable number of falls to that during LOC1 (the highest value point of the S15 group during LOC2 in Fig 4).

After R15, we did not observe ataxic and dystonic behaviors during LOC1 and LOC2. However, we noticed interindividual differences in distance traveled after treatment. During LOC1, animals 11, 18, 24, and 28 displayed no locomotor stimulation. On the contrary, animal 13 was stimulated. During LOC2, stimulation increased in some animals (animals 1, 11, 13, and 18, dotted lines) and decreased in others (animals 5, 9, 24).

Moreover, after treatment with RS30, we observed more locomotor stimulation during LOC1 in animals 42 and 44 than other animals from that group. During LOC2, we noticed fewer interindividual differences. Lastly, we did not observe large interindividual differences in groups receiving S5, R5, or saline. The exception was animal 26 receiving S5. It displayed stronger ataxia than other animals from that group (highest dot in Fig 4).
